# Physiological Anatomy and Physiology of Man, with Numerous Original Illustrations

**Published:** 1848-02

**Authors:** 


### PART II.—REVIEWS.

### ARTICLE VII.

*Physiological Anatomy and Physiology of Man, with numerous original illustrations.* By B. R. Todd, M. D., F. R. S., and W. Bowman, F. R. S., of King's College, London. To be completed in four parts, forming two volumes.

This most excellent work now being re-printed in this country, by the enterprising publishers, Lea & Blanchard, affords to the practitioner and student of medicine the best, if not the only means, of becoming acquainted with the numerous and important advances which have been made during the last five years, in general anatomy and physiology.

It is highly creditable to the authors of this work, that, to the exclusion of theory and speculation, known anatomical facts, many of which are the fruits of their own careful and laborious investigations in microscopic anatomy, are made to serve as the foundations for most of their physiological deductions.

"The study of anatomy," say they, "must always accompany that of physiology, on the principle that we must understand the construction of a machine before we can comprehend the way in which it works. The history of physiology shows that it made no advance until the progress of anatomical knowledge had unfolded the structure of the body. There is so much obvious mechanical design in the intimate structure of the various textures and organs that the discovery of that structure opens the most direct road to the determination of their uses."

The course pursued throughout the work is strictly in accordance with this view. A full description of the minute structure, constitution, and development of an organ or tissue, precedes, always, and is made the basis of conclusions with regard to its functions; physiological views of their own, and of others, are fairly and candidly given, but none are recommended as worthy of being received and adopted, excepting such as are strictly in accordance with anatomical facts.

By pursuing such a course, (in our opinion the only proper one,) much additional information, both with regard to structure and functions of the animal organization, has been acquired, errors have been detected, and plausible theoretical views, not based on facts, have been made to appear less attractive.

The first eight chapters of the work treat of motion, and afford ample evidence of the ability of the authors to do justice to any subject.

It is well known to our readers that a few years since it was discovered that nearly all fluids contained moving particles, and that these were described, by several observers, as living animals, liable to be slain by thousands, whenever a man saw fit to quench his thirst with a glass of cold water.

The following quotation may serve to enlighten the credulous, and to quiet the consciences of the humane:

"The term *molecular motion* was used many years ago by Mr. Robert Brown, to denote a phenomenon which he had witnessed in the particles of various organic and inorganic substances in a state of extremely minute subdivision. When these particles were suspended in water, they exhibited, under the microscope, motions, which consisted in more or less rapid oscillations and rotations of the particles themselves. He found them in the pollen of plants, in many mineral and metallic substances, in various animal matters, reducedto a subtle powder, consisting of particles that ranged in diameter between the 1-15000th and 1-30000th of an inch. The movements are clearly not peculiar to living or organic parts, for they occur in inanimate ones: they never occur excepting when the particles are suspended in water, or some liquid; and they are attributable to currents produced in the fluid by evaporation at its surface or edges, for they may be arrested by covering the fluid with oil, or using other means to prevent such evaporation. They are not, therefore, inherent in the particles themselves, which only obey the impulse communicated to them by the currents created in the fluid which holds them in suspension."

On cilia and the cause of ciliary motion our authors make the following remarks :

"Certain surfaces, which are, in their natural and healthy state, lubricated by fluid, are covered with a multitude of hair-like processes, of extreme delicacy of structure and minuteness of size. These are called *cilia*, from *cilium*, an eyelash. They are generally conical in shape, being attached by their bases to the epithelium that covers the surface on which they play, and tapering gradually to a point; or, as Purkinje and Valentin state, they are more or less flattened processes, of which the free extremities are rounded; and this latter form prevails in the human subject.

They vary in length from the 1-1000 to the 1-12000 of an inch. They are disposed in rows, and are adapted in their arrangement to the shape and extent of the surface to which they belong; they adhere to the edges, or to a portion of the surface, of the particles, of the epithelium, preferring the columnar variety of them.

During life, and for a certain period after death, these filaments exhibit a remarkable movement of a fanning or a lashing kind, so that each cilium bends rapidly in one direction, and returns again to the quiescent state. The motion, when viewed under a high magnifying power, is singularly beautiful, presenting an appearance somewhat resembling that of a field of corn agitated by a steady breeze. Any minute objects coming in contact with the free extremities of the cilia are hurried rapidly along in the direction of the predominant movement; one or more blood-discs, accidentally present, will sometimes pass rapidly across the field, propelled in this way, and very minute particles of powered charcoal may be conveniently used to exhibit this phenomenon, and to indicate the direction of the movement. The action of the cilia produces a current in the surrounding fluid, the direction of which is shown by the course which the propelled particles take.

An easy way to observe this phenomenon is to detach by scraping with a knife a few scales of epithelium from the back of the throat of a living frog. These, moistened with water, or serum, will continue to exhibit the movement of their adherent cilia for a very considerable time, provided the piece be kept duly moistened. On one occasion we observed a piece prepared in this way exhibit motion for seventeen hours; and it would probably have continued doing so for a longer time, had not the moisture around it evaporated. However, Purkinje and Valentin have observed it to last for a much longer time than this in connection with the body of the animal. In the turtle, after death by decapitation, they found it lasted, in the mouth, nine days; in the trachea and the lungs, thirteen days; and, in the œsophagus, nineteen days. In frogs, from which the brain had been removed, it lasted from four to five days. The longest time they observed it to continue in man and mammalia was two days; but in general it did not last nearly so long. What appears to be immediately necessary to the continuation of the movement, is the integrity of the epithelial cells to which the cilia adhere; for as soon as these shrink up for want of moisture, or become physically altered by chemical reagents or by the progress of putrefaction, the cilia immediately cease to play.

From these facts we learn two important points in connection with this phenomenon. The first is, the truly molecular character of the movement. Whatever be the immediate cause of the action of the cilia, it is evidently intimately connected with the minute epithelial particles to which they are attached; for cilia never exist in man and the higher animals without epithelial particles, and these particles have no organic connection with the subjacent textures, excepting such as may arise from simple adhesion. And, secondly, we perceive, that this movement is independent of both the vascular and the nervous systems, for it will continue to manifest itself for many hours in a single particle isolated from the rest of the system. After death it remains longer than the contractility of muscle; a circumstance which, together with the facts just mentioned, indicates that the cilia cannot be moved by little muscles inserted into their bases, as some have supposed. And experiment also shows this independence. If the abdominal aorta be tied, the muscles of the lower extremities will be paralyzed in consequence of their being deprived of their blood; and on removing the ligature, and allowing the blood to flow, the muscles wil recover themselves. But a ciliated surface is not affected at all in its movements, though the supply of blood to the subjacent tissues be completely cut off. Again, hydrocyanic acid, opium, strychnia, belladonna, substances which exert a powerful effect on the nervous system, produce no influence upon ciliary motion. In the bodies of animals killed by these poisons, the phenomenon is still conspicuous; and even the local application of them does not hinder it, provided the solutions do not injure the epithelial texture. Shocks of electricity passed through the ciliated parts, do not affect the movement. Lastly, the removal of the brain and spinal cord in frogs, by which all muscular movements are destroyed, does not stop the action of the cilia. This striking fact may likewise be adduced to disprove the supposition, that these movements result from the action of minute muscles; for, although muscles may be excited to contract without nerves, we have no instances in the higher animals in which they habitually act without the interference of the nervous system; nor is it likely that a movement existing over so extended a surface, as that by the cilia, would, if effected by muscles, be independent of nervous influence.

What is the cause of ciliary motion? We have shown it to be independent of the blood and of the nerves, and to resist those depressing causes which usually put a stop to the action of contractile tissue. It requires for its continuance three conditions: a perfect epithelium cell; moisture, not of too great density; and a temperature within certain limits. From Schwann's observations it appears that cells exhibit a power of endosmose; that a chemical change occurs in the fluids in contact with them; and that a movement of their internal granules may be seen under certain circumstances. If ciliated epithelium cells exert an attraction of endosmose upon the surrounding fluid, may not this physical phenomenon afford a clue to determine the cause of the movement."

The recently developed facts concerning the structure of bones, and especially with regard to the manner in which their nutrition is provided for, are among the most interesting.

The vascular membranes to which the blood vessels for the nutrition of bone are distributed, are the periosteum upon the surface, the medullary membrane lining the large cavities, and the reflections from both of these into the Haversian canals. Intervening between these membranes and the surfaces, both external and internal, with which they lie in contact is found a layer of nucleated cells with a basement membrane, by means of which the nutritious material is withdrawn from the capillaries, and transmitted to the bony structure composed of granules and a beautiful system of pores and cavities, described in the following extract:

"The most interesting points in the minute anatomy of bone relate to the mode in which nutrition is provided for in those parts not in immediate contact with the blood-vessels. We have already seen that considerable masses of cartilage derive their nutriment from vessels placed on their exterior only, apparently by a kind of imbibition, perhaps aided by the presence of the nucleated cells, and by a more or less fibrous texture: but bone, which is of a far harder and denser nature, is unable to imbibe its nourishment so easily. Hence its surface is greatly augmented by the arrangements already detailed; and, in addition to this, the osseous tissue itself is provided with a special system of microscopic cavities and canaliculi, or pores, by which its recesses may be irrigated, to a degree of minuteness greatly exceeding what could have been effected by blood-vessels alone, consistently with the compactness and density required in the tissue. The study of this delicate apparatus will now demand attention, but a few words must be premised on the ultimate structure of the *osseous tissue.*

It appears from the researches of Mr. Tomes, about to be published in the Cyclopædia of Anatomy, that the ultimate structure of the osseous tissue is *granular.* The granules of bone are often very distinctly visible, without any artificial preparation, in the substance of the delicate spiculæ of the cancelli, viewed with a high power, and in various sections of all forms of bone.

Where bone exists naturally in an exceedingly attenuated form it may consist of a mere aggregation of these granules, unpenetrated by any perceptible pores. This constitutes the simplest form under which the tissue can present itself.

But all the osseous tissue with which the human anatomist is concerned is of such bulk as to contain the series of pores and cavities already alluded to for the conveyance of fluid from and to its vascular surface. These *pores* always advance into the bone from open orifices on its surface. They soon arrange themselves in sets, each of which, after anastomosing with neighboring ones, discharges itself into a small cavity or *lacuna*, in which its individual pores coalesce. From the sides of this lacuna other pores pass off to similar cavities in the vicinity, and others proceed from its opposite surface to penetrate still deeper into the tissue. These pour themselves into another lacuna, or divide themselves between two or three, which are connected in like manner by lateral channels. From these again pass others, which pursue an onward course from the surface; and so on, until the whole substance of the bone is perforated by them. The pores from the further side of the extreme lacunæ either open on the surface of the bone which they may now have reached, or else take a recurved direction back into the tissue.

When this beautiful system of microscopic pores and cavities was first seen, it was not recognized as such. The lacunæ were imagined to be solid *corpuscles* (a name still commonly applied to them,) and the lines radiating from them to be branching threads of the earthy constituent of bone. They may be proved in many ways, however, to be real excavations in the tissue. With a sufficiently high power their opposite walls can be distinctly seen, as well as their interior; but the most conclusive evidence lies in our being able to fill them with fluid."

The following quotation from the British and Foreign Medico-Chirurgical Review, for January, 1848, shows that interesting discoveries are still being made:

"The chief purpose of this paper is to draw attention to the differences that exist between the bones of different animals, in regard to the size and form of the osseous lacunæ, and the number and mode of radiation of their canalicula. In Mr. Quekett's opinion, these characters are sufficiently definite and constant to serve for the determination of the class, and sometimes even of the order, to which the animal belonged, from the microscopic examination of even a minute fragment of one of its bones. If this should be capable of satisfactory proof,—and we have great confidence that Mr. Quekett would not advance any general statement of this kind but as the result of sufficiently extended researches,—the test is one of great importance in palæontology, whilst the fact is one of high interest to the physiologist. We may notice that Mr. Bowerbank has arrived at the same general conclusions; and has specially applied this test to the determination of some doubtful wing-bones found at the Isle of Sheppey. The question lay between their having belonged to a long-winged sea-bird, such as the albatros, or to a gigantic pterodactyle; and it was decided, unequivocally as we believe, in favor of the latter, thus enlarging our ideas of the size of these flying lizards of the ancient world, since the creatures of which the fragments in question formed part must have had a spread of wing not less than twelve or fourteen feet."

Muscular fibres, as determined by all recent investigations, are found to be of two kinds; the striped, composing all the voluntary muscles, and found, also, in those of the pharynx and heart; and the unstriped, constituting the muscular coats of the stomach, intestines, bladder, uterus and other contractile organs and tissues not influenced by volition.

"The voluntary fibre always presents, upon and within it, longitudinal dark lines, along which it will generally split up into fibrillæ; but it is by a fracture alone that such fibrillæ are obtained. They do not exist as such in the fibre. And, further, it occasionally happens that no disposition whatever is shown to this longitudinal cleavage; but that, on the contrary, violence causes a separation along the transverse dark lines, which always intersect the fibre in a plane perpendicular to its axis. By such a cleavage, discs, and not fibrillæ, are obtained; and this cleavage is just as natural, though less frequent than the former.—Hence it is as proper to say that the fibre is a pile of discs, as that it is a bundle of fibrillæ; but, in fact, it is neither the one nor the other, but a mass in whose structure there is an intimation of the existence of both, and a tendency to cleave in the two directions. If there were a general dis integration along all the lines in both direction, there would result a series of particles, which may be termed *primitive particles* or *sarcous elements*, the union of which constitutes the mass of the fibre. These elementary particles are arranged and united together in the two directions. All the resulting discs as well as fibrillæ are equal to one another in size, and contain an equal number of particles. The same particles compose both. To detach an entire fibrillæ is to abstract a particle of every disc, and *vice versa*. The width of the fibre is therefore uniform, and is equal to the diameter of any one of the discs. Its length is the length of any one of its fibrillæ, and is liable to the greatest variety.

"The striped fibre is enclosed in a tubular sheath or *sarcolemma*, adapted to its surface, and adhering to it. This consists of a transparent, very delicate, but tough and elastic membrane, which isolates the fibre from all other tissues. In general, it has no appearance of any kind of structure; but in the case of bulky fibres, where it is strong in proportion, faint indications may be detected of a complex interweaving of filaments far too minute to be individually recognized. It occasionally has small corpuscles, the remains of cell-nuclei, in contact with it.

"Every fibre is attached by its extremities to fibrous tissue, or to some tissue analogous to it ; but an accurate examination of this difficult subject lends no countenance to the ordinary received opinion, that the tissue is prolonged over the whole fibre from end to end, as its cellular sheath ; nor is this view reconcilable with the physical requirements of the case. It is extremely difficult to isolate a muscular fibre, with the tendinous fibrillæ pertaining to it, either in mammalia or birds ; but this may be occasionally accomplished in fishes, and in certain muscles of insects. In these examples, the minute detachment of the fibrous tissue may be seen to pass, and to become attached to the truncated extremity of the fibre. The fibre ends by a perfect disc, and with the whole surface of this disc the tendon is connected and continuous. The sarcolemma ceases abruptly at the circumference of the terminal disc, and here some small part of the tendinous material appears to be joined to it.

"Muscles grow by an increase, not of the number, but of the bulk of their elementary fibres : there is reason to believe that the number of fibres remain through life as it was in the fœtus, and that the spare or muscular build of the individual is determined by the mould in which his body was originally cast."

The unstriped fibres "consist of flattened bands, generally of a pale color, bulged at frequent intervals by elongated puscles, similar to those of striped muscle and capable of being displayed by the same process. The texture of these fibres seem to be homogeneous. By transmitted light, they have usually a soft, very finely mottled aspect, and without a darkly-shaded border. Sometimes the mottling is so decided as to appear granular, and occasionally these granules are arranged in a linear series for some distance. This condition is probably an approach towards the structure of the striped fibre, for these granules are about the size of the sarcous elements already described."

Muscles receive their nutrition from capillary vessels of a size to admit of the passage of but a single row of blood globules, and consisting of "longitudinal and transverse vessels : the longitudinal always following the course of the elementary fibres, and lying in the intervals between them ; the transverse being short communications placed at nearly equal distances between the longitudinal ones. and crossing nearly, or quite, transversely over or under the fibres."

Nerves distributed to muscles are found to pass, like blood vessels, between the primitive fibres and to cross them not transversely, like the capillaries, but obliquely ; the ultimate tubules of the nerves, separating into sets of two or more and ultimately from each other, forming arches and returning either to another or to the same trunk from which they set out.

"In this loop-like course they accompany to some extent the minute blood-vessels, but do not accurately follow them in their last windings, since their distribution is in a different figure. They pass among the fibres of the muscle, and touch the sarcolemma as they pass ; but, as far as present researches have informed us they are entirely precluded by this structure from all contact with the contratile material, and from all immediate intercourse with it. How then shall we explain the transmission of the nervous influence to a material thus enclosed? If it were wise or safe to go a single step in advance of pure observation on so obtruse a question, we might suggest, resting on the seemingly sure ground of exact anatomy, that this influence must be of a nature capable of emanating beyond the limits of the organ which furnishes it. But further than this, as to how, or to what extent this influence may so emanate, or as to what may be the nature, it would, perhaps, in the present state of knowledge, be hardly warrantable even to speculate."

For want of time and space, we are compelled to defer for the present, making quotations from other still more interesting chapters. We hope to be able to give at some future time that degree of attention which it merits to other portions of the work. H.

